# Hybridization and restricted gene flow between native and introduced stocks of Alpine whitefish (*Coregonus* sp.) across multiple environments

**DOI:** 10.1111/j.1365-294X.2010.04961.x

**Published:** 2011-02

**Authors:** Kathrin A Winkler, Barbara Pamminger-Lahnsteiner, Josef Wanzenböck, Steven Weiss

**Affiliations:** * Institute for Zoology, Karl-Franzens University Graz, Universitätsplatz 2A-8010 Graz, Austria; † Institute for Limnology, Austrian Academy of SciencesMondseestraße 9, A-5310 Mondsee, Austria

**Keywords:** admixture, artificial hybrid zones, Austria, introgression, stocking

## Abstract

Translocations of Baltic whitefish (*Coregonus* sp.) into Austrian Alpine lakes have created ‘artificial hybrid zones’, threatening the genetic integrity of native lineages. We evaluate the genetic structure of *Coregonus* in Austrian lakes and characterize hybridization and introgression between native and introduced lineages. Fifteen populations (*N*= 747) were assessed for allelic variation at eight microsatellite loci and a reduced set (*N*= 253) for variation across two mtDNA genes (cyt *b* and NADH-3). Bayesian approaches were used to estimate individual admixture proportions (*q*-values) and classify genotypes as native, introduced or hybrids. *q*-value distributions varied among populations highlighting differential hybridization and introgression histories. Many lakes revealed a clear distinction between native and introduced genotypes despite hybridization, whereas some locations revealed hybrid swarms. Genetic structure among lakes was congruent with morphological divergence and novelty raising speculation of multiple taxa, including a population south of the Alps, outside the putative native range of *Coregonus*. Although statistically congruent with inferences based on nuclear markers, mitochondrial haplotype data was not diagnostic with respect to native and non-native lineages, supporting that the Alpine region was colonized post-glacially by an admixture of mtDNA lineages, which coalesce >1 Ma. Mechanisms promoting or eroding lineage isolation are discussed, as well as a high potential to conserve native Alpine lineages despite the extensive historical use of introduced Baltic stocks.

## Introduction

Increasing habitat degradation and translocation of plants and animals has been linked to increasing rates of hybridization. The consequences of such anthropogenic hybridization are of high interest to conservation when introgression with a non-native or domesticated lineage threatens the existence or genetic integrity of native populations in the wild (Kidd *et al.* 2009; Muhlfeld *et al.* 2009; Ryan *et al.* 2009). Allendorf *et al.* (2001) recognized three categories of anthropogenic hybridization: (1) hybridization without introgression, (2) widespread introgression, and (3) complete admixture. Each of these categories raises different conservation concerns as well as methodological challenges. Hybridization without introgression is not a genetic issue, but nonetheless can seriously affect the native species of interest when hybrids are frequent or the population is small as hybrids represent wasted reproductive effort (Allendorf *et al.* 2001). To move beyond this first category, the presence and degree of introgression must be determined. The pattern of hybridization and introgression may reflect a variety of environmental and biological factors that determine if and to what extent an invasive species will be successful, and thus threatens the existence of native gene pools. Such conservation genetic dilemmas frequently involve salmonid fishes, which attract the incessant attention of resource managers with real or perceived problems with respect to the demands of both commercial and recreational fisheries (Leary *et al.* 1995; Rubidge & Taylor 2004). Whitefishes (*Coregonus*) are a typical example among salmonid fishes subject to intensive management as well as conservation concerns due to threats associated with introduced or domesticated lineages.

European whitefishes (*C. lavaretus* complex) are specifically noted to have undergone rapid (post-glacial) radiation in lacustrine systems of the Central European Alps. For example, in Swiss lakes, more than two species per lake are commonly reported (Steinmann 1950; Kottelat & Freyhof 2007) and research has focused on the association of biological units to various ecological niches associated with feeding and spawning ecology (Douglas *et al.* 1999; Kahilainen & Østbye 2006; Østbye *et al.* 2006; Kahilainen *et al.* 2007; Vonlanthen *et al.* 2009). Austrian Alpine lakes on the north slopes of the Alps harbour native *Coregonus* sp. exhibiting several ecotypes, which have been recognized as different species by some authors (Neresheimer & Ruttner 1928; Benda 1949; Kottelat & Freyhoff 2007), although the intra-lake diversity has never been assumed to be as high as that found in Swiss lakes. These stocks have been subjected to a variety of management schemes involving rearing, transport and stocking (Luczynski & Ritterbusch-Nauwerck 1995) reaching back to the 16th century (Diem 1964). While some systems use their own stocks for enhancement programmes others rely on commercially available brood. ‘Maräne’, *Coregonus maraena* (Bloch 1779) were exported into Bohemian aquaculture in the 19th century from Lake Miedwie (Poland) (Šusta 1898). This Baltic stock has been extensively used in Austrian aquaculture for at least 50 years and is a primary source for whitefish stock enhancement (Jagsch 1992). *C. maraena* from Lake Miedwie were extirpated hindering any attempt to compare the genetic integrity of today’s aquacultural strain with their historical source. Presently, some authors consider a range of stocks in the Baltic Sea to comprise *C. maraena* (Kottelat & Freyhof 2007) without regard to, or comparison with the commercially available hatchery strain.

Comprehensive study of the phenotypic and genetic structure of *Coregonus* in Austria has never been carried out. Because the hatchery Maräne are extensively used in Austrian management regimes, it is hypothesized that the original genetic integrity of native whitefish populations has been lost and this view supports neglect concerning the conservation of native populations (Luczynski & Ritterbusch-Nauwerck 1995; Ritterbusch-Nauwerck & Lahnsteiner 2005). Lahnsteiner & Wanzenböck (2004) and Wanzenböck *et al.* (in press) have provided both empirical and experimental evidence for pre-zygotic isolating mechanisms in the form of temporal, if not spatio-temporal spawning segregation between native and introduced stocks of Austrian whitefish. While the generality of these observations has not been elaborated, it suggests that there is a potential for the conservation of native populations.

Clarification of the pattern and extent of hybridization and introgression among Alpine lakes as well as evaluation of the among lake structure and diversity should support theoretical inferences on the consequences of future introductions, as well as practical conclusions concerning the feasibility of management measures geared toward conserving the remaining genetic integrity of native stocks.

We provide the first view of the population genetic structure of Austrian *Coregonus* and examine the patterns of hybridization and introgression between the introduced and native stocks. We hypothesize that the introduced Baltic *Coregonus* does not freely hybridize and produce hybrid swarms with native Alpine stocks. Rather, we suggest that one or more mechanisms (spatial, temporal or relating to intrinsic reproductive compatibility) promote varying degrees of reproductive isolation. We augment our genetic data with measurements of quantitative morphological traits both to raise the resolution in detecting introgression and to ask if there is a quantitative phenotypic affect associated with varying levels of introgression. We further speculate that historical ecological deterioration and concomitant stock collapse may have left lakes more vulnerable to introgression with Maräne than lakes that have remained ecologically stable. Additionally, we draw further inferences on the evolutionary history of the native stocks and discuss the implications of such results for conservation planning.

Due to the inconsistent and controversial taxonomic nomenclature applied to European *Coregonus*, we use an ‘operational unit’ (OU) approach with no *a-priori* assumption of the number of biological units involved. Accordingly *Coregonus* stocks were classified as either OU-native or OU-introduced.

## Materials and methods

### Sampling and DNA extraction

**Fig. 1:**
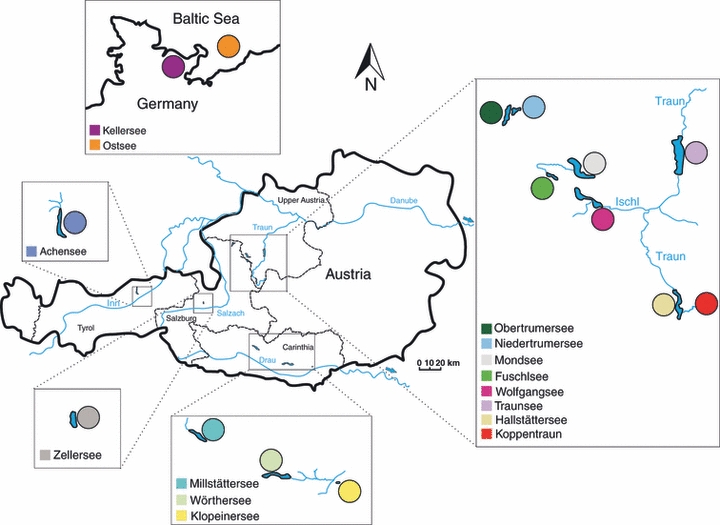
Sampled populations of *Coregonus* (primarily in Austria). Sample sites are colour coded for subsequent cross-referencing.

Adult fish were sampled from 12 Austrian lakes using monofilament gillnets. All Austrian populations belong to the Danube drainage, and to our knowledge only one of them (Achensee) has been integrated into previous studies (Douglas & Brunner 2002). Three additional samples of Baltic origin included the so-called ‘hatchery Maräne’ (hence referred to as OU-introduced) and two wild-caught samples from coastal waters in Germany and Poland (Table 1; Fig. 1). The OU-introduced is a commercial stock reared for decades in the Waldviertel region of Austria and widely used for management throughout the country. Larvae from one additional Austrian lake (Traunsee) were included for some limited genetic analysis (mtDNA). Fin clips were preserved in 95% ethanol and total genomic DNA was extracted using a high-salt (ammonium acetate) extraction protocol modified from Miller *et al.* (1988).

**Table 1:** Sample locations and information on native status, stocking management and ecological degradation. This latter rating is compiled from data on nutrient loads, secchi disk depths and oxygen levels during the peak of degradation in the mid-1970s, drawn largely from Sampl *et al.* (1982)

Lakes	Sample code	Native stock	Stocked	Source of fish brood	Latitude	Longitude	Maximum depth [m]	Ecological degradation (eutrophication)
Fuschlsee	FUS	no	yes	Hallstätter-and Attersee, Maräne*	47°48′	13°16′	67	low
Hallstättersee	HAL	yes	yes	Maräne*	47°34′	13°39′	125	medium
Koppentraun^1^	KOP	yes	no	n.a	–	–	–	n.a
Mondsee	MON	yes	yes	Maräne*	47°51′	13°21′	68	medium
Niedertrumersee	NIE	yes	yes	Maräne*	47°59′	13°07′	42	medium
Obertrumersee	OBE	yes	yes	Maräne*	47°57′	13°04′	36	heavy
Traunsee	TRA	yes	yes	Bodensee, Traunsee	48°31′	15°15′	191	low
Wolfgangsee	WOL	yes	yes	Maräne*	46°38′	14°09′	113	low
Zellersee	ZEL	yes	yes	Hallstätter-, Atter-, Boden-,	47°19′	12°48′	69	heavy^3^
				Wolfgangsee, Maräne*				
Klopeinersee	KLO	?	no	n.a	46°36′	14°34′	48	heavy
Millstättersee	MIL	?	yes	Hallstätter- and Mondsee, Maräne*	46°47′	13°34′	141	heavy
Wörthersee	WOE	?	yes	Maräne*	47°45′	13°23′	85	heavy
Achensee	ACH	yes	yes	Bodensee (sporadic events in the seventies)	47°26′	11°43′	133	low
Kellersee	KEL	n.a	n.a	n.a	54°10′	10°35′	27	n.a
Ostsee	OST	n.a	n.a	n.a	59°00	21°00′	n.a	n.a
Waldviertel^2^	WAL	n.a	n.a	Maräne*	n.a	n.a	n.a	n.a

n.a: not applicable

* All so called “Maräne” (OU-introduced) stem from pond aquaculture from the Waldviertel region, and originally from imported material.

? These sample sites are located in the Province of Carinthia, south of the Alps.

1 Main tributary to Hallstättersee.

2 Waldviertel is a region of northeast Austria where pond aquaculture with Maräne has been carried out for at least 50 years.

3 Ecological degradation did not stem from eutrophication but rather due to middle-ages mining activities associated with strong fish kills.

### Microsatellite genotyping and genetic diversity

A total of 747 individuals from 14 sample locations and 1 reference stock were screened for allelic variation at 8 tetranucelotide microsatellite loci cloned from Austrian *Coregonus*: *ClaTet1*, *ClaTet5, ClaTet6*, *ClaTet9*, *ClaTet12*, *ClaTet13*, *ClaTet15* and *ClaTet18* (Winkler & Weiss 2008). These eight loci were chosen from those reported in Winkler & Weiss (2008) based on their high information content and multiplex compatibility. PCR conditions and genotyping procedures are reported in Winkler & Weiss (2008). The number of alleles per locus (*AN*) and the observed and expected heterozygosity (*H*_O_, *H*_E_) was calculated using genetix software 4.05 (Belkhir *et al.* 1996-2004). Allelic richness per locus (*AR*) as well as deviations from Hardy–Weinberg equilibrium (*F*_IS_ per population) was calculated using fstat version 2.9.3.2 (Goudet 2001). The software Micro-Checker (van Oosterhout *et al.* 2004) was used to assess the potential presence of null alleles. Tests for linkage disequilibrium (LD) at both global and population level were performed with fstat using the default Markov chain parameters.

We analysed population structure at four distinct levels in order to maximize resolution dependent on the question. First (a) we attempted to identify and classify OU-native, OU-introduced and hybrids at the global scale; second (b) we further evaluated the potential number of genetic units within the OU-native taxon at the global scale; third (c) we tested for potential multiple genetic units within each lake; and finally (d) we evaluated the pattern of introgression within each lake.

#### Identification and classification of OU-native, OU-introduced and hybrids

For identification of OU-native, OU-introduced and hybrid individuals a model-based clustering method was used as implemented in structure 2.1 (Pritchard *et al.* 2000). An admixture model assuming independent allele frequencies with a burn-in of 50 000 followed by 100 000 iterations was applied. Setting *K*= 15 or *K*= 13, (i.e. with or without the two Baltic samples, OST and KEL) five independent Markov chain Monte Carlo (MCMC) simulations were run. We derived the most likely value of *K* using the second-order rate of change *L*′′ (*K*) following Evanno *et al.* (2005) using the online tool Structure Harvester (http://taylor0.biology.ucla.edu/struct_harvest). Following this approach the most likely population structure consisted of two clusters, reflecting our expectations of one divergent introduced (Baltic) lineage and one or more native lineages. Thus, for subsequent assignment of individuals to pure and hybrid categories, *K* was set to 2. For comparison, we applied a second Bayesian clustering approach as implemented in newhybrids ver. 1.1 (Anderson & Thompson 2002), applying the same MCMC conditions and criteria as with structure, including five replicates. newhybrids assumes that the sample consists of a mixture of pure individuals and hybrids, and each individual is assigned to one of several different hybrid categories (i.e. F1 and F2 hybrids and the first two backcross classes). However, considering the number of loci applied (Vähä & Primmer 2006) we used a so-called ‘relaxed’ option and only attempted to categorize individuals as pure-bred or ‘hybrid’, whereby hybrid was understood as any of the four hybrid categories named in newhybrids. The setting of threshold posterior probability values for assignment of individuals to purebred or hybrid categories involves a trade-off between accuracy and efficiency (Vähä & Primmer 2006). For both structure and newhybrids we used a relatively stringent posterior probability threshold (0.90), which minimizes the number of misidentified pure-bred fish while maximizing the efficiency of assigning hybrids (Vähä & Primmer 2006; Burgarella *et al.* 2009). This choice allows us to more confidently describe the genetic structure and phenotypic diversity of the OU-native lineage as well as its genetic differentiation from the OU-introduced. We additionally emphasize that our main interest in evaluating *q*-value distributions among lakes is not affected by thresholds. Factorial correspondence analysis (FCA) of diploid genetic data can be carried out based on the principles outlined in She *et al.* (1987) in the software package genetix 4.05. We depicted the first two axis of an FCA on individual genotypes, whereby each data point was colour-coded according to the assignment categories produced in our structure analysis. This depiction was repeated for a more stringent (0.90) and less stringent (0.75) assignment threshold.

#### Global structure of OU-native

Individuals displaying threshold *q*-values of >0.90 (according to structure) were used to evaluate patterns of genetic variation within the indigenous gene pool. Among population differentiation was quantified using *F*_ST_ and *R*_ST_ as calculated in fstat and Arlequin ver. 3.1.1 (Excoffier *et al.* 2005). Statistical significance was determined using 78 000 (fstat) and 10 100 (Arlequin) permutations. Neighbour-Joining trees of populations, based on the model-free shared allele distances (*D*_AS_), were constructed with the program populations 1.2.30 (Langella 2002). This distance is recommended for closely related populations as well as for microsatellite data in general (Nei *et al.* 1983; Takezaki & Nei 1996). Node support was generated through bootstrapping across loci (1000 replicates).

#### Structure of OU-native within individual lakes

To test for the potential of within lake structure of the OU-native we applied structure analysis to each lake individually, again, after removing the OU-introduced taxon as well as hybrids based on a q-threshold of 0.90. The same structure conditions as above were used whereby *K* was set to three, given that more than two *Coregonus* taxa have never been reported from Austrian lakes.

#### Population specific levels of hybridization and comparison to simulations

To display the pattern of lake-specific introgression we used the mean *q*-values from five replicates (*K*= 2) of the global structure analysis (12 lakes and the WAL reference). We additionally produced simulated hybrid populations and compared their *q*-value distributions to those in actual lakes to gain confidence in our lake-specific inferences. Synthetic hybrids were created using hybridlab 1.0 (Nielsen *et al.* 2006) based on reference populations from both the OU-native (represented by HAL and KOP) and the OU-introduced (WAL). Two different sets of synthetic hybrids were produced, one based on the full sample size of the reference populations (*N*= 102 for each), and a second based on a reduced set of individuals (*N*= 79 for each) using the *q*-threshold of 0.90 (see results below). For both simulations, 100 synthetic genotypes were generated.

### mtDNA analyses

To allow broader-scale inferences concerning our lakes and to compare them with existing phylogeographic information we also analysed mitochondrial DNA (mtDNA) for a reduced set (*N*= 253) of our samples. MtDNA sequence variation was analysed for these individuals across segments of the cytochrome oxidase *b* (cyt *b*) and the NADH-3 subunit (ND3) as described in Østbye *et al.* (2005). PCR products were purified using ExoSAP-IT and sequenced (BigDye, ABI PRISM 3130xl capillary genotyper). Mitochondrial DNA sequences from both fragments were edited, combined, and aligned with 59 published haplotpyes (Østbye *et al.* 2005) using mega 4.1 (Tamura *et al.* 2007). Potential linkage between microsatellite alleles and mtDNA was tested using fstat whereby individual haplotypes were re-coded as dummy alleles representing major mtDNA clades.

To evaluate the within and among lake distribution of closely related haplotypes a median joining network was obtained with network 4.5.1.0 (Bandelt *et al.* 1999). Differences among haplotype diversity between taxa were assessed with a Fisher’s exact test. Time to the most recent common ancestor (TMRCA) was calculated with the software package beast 1.5.1 (Drummond & Rambaut 2007, 2009). We used an un-partitioned dataset and a TrN+G substitution model based on a hierarchical likelihood ratio test produced in modeltest 3.06 (Posada & Crandall 1998). We used a substitution rate of 1 × 10^−8^ per site per year, equivalent to 1% divergence per million years, deemed appropriate and compatible with calibrations for salmonid fishes (see discussions in Weiss *et al.* 2002 and Froufe *et al.* 2005). We implemented constant population size and compared results using a strict molecular clock and an uncorrelated lognormal relaxed molecular clock (Drummond *et al.* 2006). An MCMC approach was adapted to criteria documented in the software manual, implicating an effective sample size (ESS) of >200 for each trace (parameter). Three independent runs (90 million generations) were carried out to ensure repeatability whereby 10% of each run was removed as an appropriate burn-in and combined in LogCombiner (beast software package). The software tracer (Drummond *et al.* 2005) was used to visualize the combined files.

### Phenotypic analysis

We used 10 morphological and 9 meristic measurements (Fig. S1, Supporting information) to complement genetic data and to evaluate the affects of hybridization and introgression characterizing the among lake diversity of the OU-native. Principal component analysis (PCA) on meristic and size-standardized morphological measurements was carried out in spss 16.0. Potential allometric effects were evaluated by regressing each morphological measurement as well as PCA factor on fish length. Mean standardized PCA factor scores for each population were used to calculate pairwise Euclidean distances between populations and to construct a UPGMA dendrogram using the program past (Hammer *et al.* 2001). This analysis included OU-native, the WAL hatchery reference and one wild-caught Baltic population (OST) for outgroup comparison. The morphological distance matrix of the ingroup was further compared to several genetic distance matrices using a mantel test with a non-parametric Spearman’s rank correlation coefficient, whereby statistical significance was evaluated with 9999 permutations using the R-package (Casgrain & Legendre 2001). These analyses were repeated with two thresholds (0.90 and 0.75) for definition of OU-native individuals.

Because our eight-locus microsatellite characterization may not detect minor levels of introgression at the individual level, we further incorporated morphological information as a surrogate for quantitative traits, to evaluate the potential effects of introgression on the native stocks. Accordingly, we evaluated the correlation between the mean lake admixture proportion (*q*-values) and the coefficient of variation (CV) for 10 morphological (i.e. ratio-variables) traits. The expectation is that the variance in these quantitative traits should be positively correlated to an increase in additive genetic variance due to introgression, reflected by higher admixture proportions of the OU-introduced lineage. As we were not interested in accounting for the obvious increased population phenotypic variance due to the inclusion of genetically recognized introduced or hybrid individuals, CVs for only OU-native individuals were used, whereby the analysis was repeated for two different thresholds as above.

## Results

### Microsatellites

Allelic variation (*AN*) was high with 17 (*ClaTet*13, *ClaTet*18) to 56 (*ClaTet*12) alleles per locus, with mean allelic diversities across lakes ranging from 6.3 (KLO) to 14.5 (MIL) (Table S1, Supporting information). Observed and expected heterozygosities ranged from 0.596 to 0.823 and from 0.565 to 0.800, respectively (Table S1, Supporting information). Each locus deviated from Hardy–Weinberg equilibrium (HWE) in one or more lakes, except *ClaTet*1, but no locus deviated significantly from HWE in all samples (Table S1, Supporting information). Across all loci, only two lakes (MON and WOE) significantly departed from HWE. There was no evidence of large allele dropout or scoring errors whereas there was evidence of null alleles due to homozygote excess, concerning the most polymorphic loci *ClaTet*6 and *ClaTet*12. However, no locus showed evidence for null alleles across all populations, and we presume that the reduced observed heterozygosities in MON and WOE were the result of a Wahlund effect stemming from the presence of both native and introduced lineages (see below). Analysis of linkage disequilibrium (LD) for the global data set revealed six highly significant comparisons out of 28. However, after removal of the OU-introduced (see identification criteria below) no evidence of LD for any locus was observed, and thus the signal of LD presumably reflects recent genetic admixture between two divergent gene pools. At the population level, signals of gametic disequilibrium were limited to one or two locus pairs within FUS, two within MIL and three within MON.

### Identification and classification of OU-native, OU-introduced and hybrids

structure analyses of 13 sample locations, including all 12 Austrian lakes sampled for adults and the WAL reference revealed two primary genetic clusters congruent with our expectations of at least two taxa. This result remains whether or not the two additional Baltic lakes are included. That is, the two wild-caught Baltic samples group with our WAL reference, supporting the Baltic origin of these stocks. Individuals were assigned to these OUs based on their *q*-values (<0.10 = OU-introduced; >0.90 = OU-native and between 0.10 and 0.90 = hybrids). Overall 661 individuals from the 12 lakes and the WAL reference were assigned to one of these three groups. *q*-values from structure assigned 359 individuals to the OU-native, 123 to the OU-introduced and 179 as hybrids. newhybrids assigned a similar number of individuals to the OU-native, but assigned considerably more hybrids at the expense of OU-introduced (355 native, 34 introduced and 272 hybrids). The genetic relationship of individuals with structure assignment (using two different threshold values) was depicted with a bi-variate FCA plot (Fig. S2, Supporting information). The first axis clearly distinguishes the two OUs and their intermediate hybrids. Individuals corresponding to OU-introduced and hybrids formed relatively homogenous groups, whereas OU-native individuals were more variable, primarily due to the differentiation of the KLO population delineated along the second axis (Fig. S2, Supporting information). The higher threshold (0.90) is more efficient in identifying hybrids, but at the cost of accuracy in the sense that more individuals of ‘true’ pure-bred origin are also assigned to the hybrid class (Vähä & Primmer 2006).

### Global structure of OU-native

Individuals classified as OU-native were used to analyse the genetic structure of native stocks among lakes, whereby Baltic samples were included. Almost all pairwise comparisons revealed statistically significant levels of differentiation (Table 2). The global *R*_ST_ and *F*_ST_ values were quite similar (0.109 and 0.123) implying that non-mutational mechanisms (i.e. genetic drift and gene flow) are responsible for this level of differentiation (Estoup & Angers 1998; Hardy *et al.* 2003). However, pairwise comparisons revealed considerably higher *R*_ST_ over *F*_ST_ values for the populations KLO and ACH (Table 2) supporting that mutation has played a larger role relative to migration in the differentiation of these two populations (Hardy *et al.* 2003).

**Table 2:** Pairwise estimates of *F*_*st*_ (above diagonal) and *R*_*st*_ (below diagonal) among ‘OU-native’ populations and the OU-introduced reference (WAL) including corresponding statistical significance with a table-wide correction (*P < 0.05; **P < 0.01; ***P < 0.001)

Sample site	ACH	FUS	HAL	KOP	KLO	MON	MIL	NIE	OBE	WOE	WOL	ZEL	WAL
ACH	–	0.1537***	0.1464***	0.1604***	0.2355***	0.1664***	0.1384***	0.1917***	0.2226***	0.1567***	0.1543***	0.1585***	0.2470***
FUS	0.4947***	–	0.0883***	0.1160***	0.2222***	0.0150	0.0674***	0.1275***	0.1281***	0.0888***	0.0317	0.0702***	0.2352***
HAL	0.5079***	0.1647***	–	0.0177	0.1643***	0.1176***	0.0956***	0.0741***	0.0997***	0.0770***	0.0804***	0.0397***	0.1610***
KOP	0.4807***	0.2230***	0.0259	–	0.1871***	0.1339***	0.1168***	0.0761***	0.1007***	0.1077***	0.0973***	0.0450*	0.1782***
KLO	0.2708***	0.3895***	0.2902***	0.2584***	–	0.2656***	0.1336***	0.1685***	0.1951***	0.1261***	0.1965***	0.1549 ***	0.2295***
MON	0.5844***	−0.0339	0.1871***	0.2381***	0.4889***	–	0.1090***	0.1569***	0.1544***	0.1287***	0.0424***	0.0896 ***	0.2639***
MIL	0.3162***	0.1088**	0.2766***	0.3329***	0.2586***	0.2042***	–	0.0846***	0.0912***	0.0106	0.0586***	0.0579 ***	0.1904***
NIE	0.3795***	0.0831*	0.0092	0.0452	0.2132***	0.0764**	0.1029**	–	−0.0039	0.0496	0.1370***	0.0327	0.1749***
OBE	0.3319***	0.0038	0.0088	0.0327	0.1314**	−0.1264	0.0885**	−0.0313	–	0.0568 **	0.1569***	0.0512	0.1840***
WOE	0.2936***	0.0347	0.1841***	0.2333***	0.1385**	0.0687*	0.0156	0.0465	0.0037	–	0.0762***	0.0532 **	0.1644***
WOL	0.4837***	−0.0540	0.1415***	0.2092***	0.3360***	0.0068	0.0915**	−0.0213	−0.0895	0.0161	–	0.0701 ***	0.2310***
ZEL	0.4726***	0.0441	0.0046	0.0325	0.3154***	0.1162**	0.1123*	−0.0835	−0.1530	0.0268	0.02687	–	0.1526***
WAL	0.3534***	0.2585***	0.2385***	0.2780***	0.3107***	0.3422***	0.18644***	0.0544*	0.1646***	0.2536***	0.2947***	0.1312***	–

**Fig. 2:**
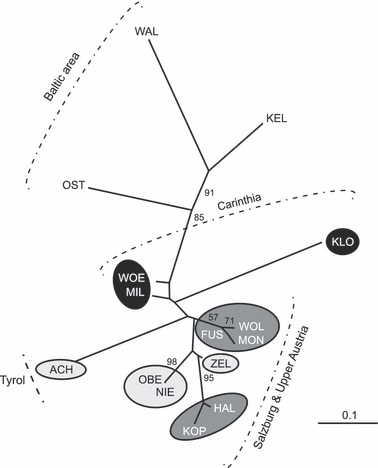
Neighbour-Joining tree of populations based on shared allele distances (*D*_AS_) for OU-native and Baltic samples. Corresponding drainage areas for OU-native populations are colour coded: black for the river Drau, dark grey for the river Traun and light grey for the river Inn. Node support values are percentages (>50%) of 1000 bootstrap replicates across loci. Abbreviations correspond to those in Table 1.

The population tree of the OU-native including the Baltic samples (KEL, OST and WAL) supported four groups of genotypes corresponding to the geographical regions (provinces), Salzburg and Upper Austria, Carinthia, Tyrol and the Baltic region, whereas the KLO and ACH populations were by far the most divergent with regard to the OU-native (Fig. 2).

### Structure of OU-native within individual lakes

Structure analysis simulations with *K*= 1 through *K*= 3 of the OU-native in each lake (*N*= 12) did not show any sign of significant population structure. Thus it can be assumed that only one native species was present in our data set for each lake.

### Population specific levels of hybridization and comparison to simulations

**Fig. 3:**
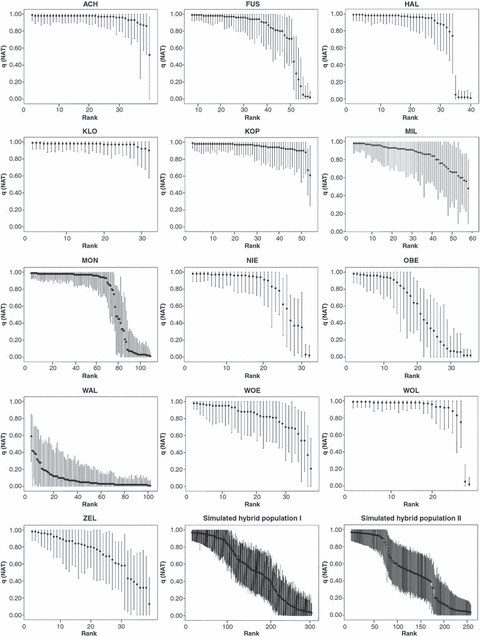
Individual admixture proportions (q-NAT) and their associated 90% credible intervals, including simulated hybrid populations. *q*-values of 1.00 represent ‘pure’ OU-native individuals and *q*-values of 0.00 imply ‘pure’ OU-introduced individuals. Values were ranked along the *x*-axis from lowest to highest. Simulated hybrid panel I is generated from parental genotypes from the whole reference samples, and panel II from parental genotypes from reference samples with a *q*-value >0.90 according to structure.

Within population structure and levels of hybridization differed among lakes (Fig. 3). The WAL reference revealed a mean *q*-value of 0.09, with 79 out of 102 individuals fulfilling the criteria of ‘pure’ OU-introduced. Simulated admixture analyses were run with both the entire WAL sample, as well as the 79 ‘pure’ individuals, though little difference was revealed (Fig. 3). Six of 12 Austrian lakes (FUS, HAL, MON, NIE, OBE and WOL) harbour both OUs albeit in varying proportions. The OU-native was most abundant ranging from 36.1 (OBE) to 100% (KLO). Pure OU*-*introduced individuals were found to a lesser extent, with the highest proportions found in OBE (22.2%) and MON (20.2%). Hybrid proportions varied from 10.3 (WOL) up to 74.3% (ZEL). Most lakes revealed an s-formed distribution of assignment (*q*-values), although with an abrupt slope clearly distinguishing the two OUs and their hybrids compared to the simulated hybrid populations (including parents). Three lakes (MIL, WOE and ZEL) exhibited a more linear pattern indicating extensive introgression with a deficiency of pure OU-introduced individuals, suggesting the result of historical rather than contemporary processes. Three lakes ACH, KLO and KOP revealed no signal of hybridization reflecting the fact that no stocking with the OU-introduced has occurred (Table 1). Mean *q*-values in these lakes ranged from 0.94 (KOP) to 0.97 (KLO).

### mtDNA analyses and correlation to microsatellite data

A total of 509 bp of mtDNA (cyt *b* and ND3 combined) was aligned and used for analyses. We found 23 different haplotypes six of which (A1, A5, A11, P1, R1 and R6) were previously described in Østbye *et al.* (2005) and 17 were novel (Table S2, Supporting information).

**Fig. 4:**
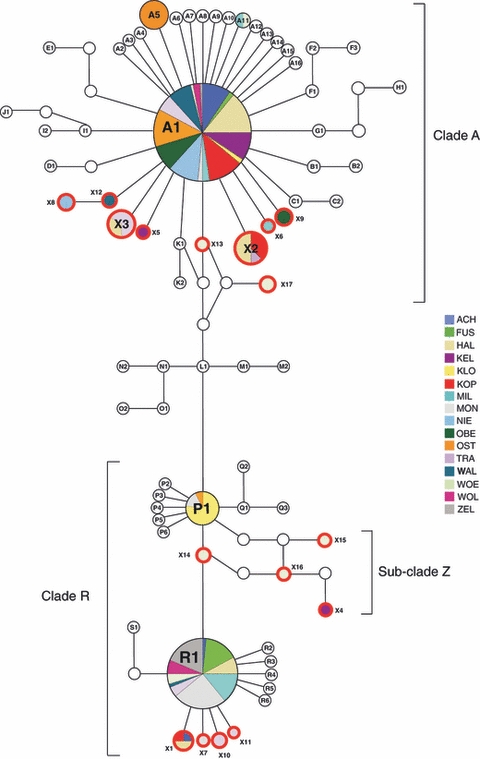
Median-Joining haplotype network of haplotypes based on concatenated mtDNA sequences (cyt *b* and ND 3), including those published in Østbye *et al.* (2005). Circles are scaled to haplotype frequency and each branch equals a single mutation regardless of length. Unlabeled circles represent theoretical haplotypes. Red circles highlight novel haplotypes. Colours correspond to sample sites following coding in Fig. 1. Haplotypes not found in this study are uncoloured.

Network analysis of all published and novel haplotypes (*N*= 76) revealed a similarly weak clade structure as shown in Østbye *et al.* (2005) (Fig. 4). The largest clade (A) comprised haplotypes mainly distributed across Northern Europe (Østbye *et al.* 2005), whereby novel haplotypes are found in two Baltic populations (X5 and X12), two south Austrian populations (X6, X13, X17) and five north Austrian populations (X2, X8, X9) (Table S3, Supporting information).

A second group (clade R) harboured haplotypes appearing in three different geographic regions: Denmark, the Baltic Sea area and the European Alps (Østbye *et al.* 2005) including novel Austrian haplotypes (X1, X7, X10, X11 and X14). A new sub-clade (Z) comprised haplotypes from one south Austrian population (X15, X16) and one Baltic population (X4).

The most common haplotypes were A1 (51%) found in each population, and R1 (25%; Fig. 4; Table S3, Supporting information). The majority of populations revealed multiple haplotypes, with TRA and WOE exhibiting the highest diversity (7 and 8 haplotypes, respectively). The minimum number of haplotypes was two, occurring in six Austrian lake populations (Table S3, Supporting information). Baltic populations (WAL, OST and KEL) revealed predominantly (90.6%) haplotypes of the Northern Europe lineage (clade A; Fig. 4). P1 haplotypes, originally reported from Denmark and the Baltic Sea area (Østbye *et al.* 2005), were predominant (83.3%) in the KLO population, although not found elsewhere in Austria except for two MON individuals classified as OU-introduced based on microsatellite analysis.

Using the microsatellite assignment-based categorization, the distribution of the main mtDNA clades (A, R and sub-clade Z) among OU-introduced, OU-native and their hybrids differed strongly (Table 3; Fisher’s exact test, *P*< 0.001). The OU-native lineage carried all clades, whereby the OU-introduced lacked haplotypes from sub-clade Z and carried a low proportion from clade R (7.7%). The highest disparity between lineages was seen for clade R, which was very frequent (43.4%) in the OU-native and carried by only three individuals of the OU-introduced. Much more than half of all hybrids carried a haplotype from clade A (63.1%), with the remaining from clade R (35.4%) and sub-clade Z (1.5%). There was no significant linkage between mtDNA clades and microsatellite allele diversity, whereby only a reduced set (*N*= 203) of the data could be tested.

**Table 3:** Haplotype frequencies (main and sub-clades) in each sample site following the network presented in Figure 4. Individuals are grouped into OU-native, OU-introduced and hybrid classes based on threshold q-values as described above

	Genetic assignment									
	
	OU-native			OU-introduced			Hybrid			
				
	Clade/sub-clade			Clade/sub-clade			Clade/sub-clade			
				
Sample site	A	R	Z	A	R	Z	A	R	Z	Total
ACH	12	2	–	–	–	–	–	–	–	14
FUS	–	8	–	2	–	–	1	2	–	13
HAL	5	1	–	3	–	–	2	–	–	11
KEL	n.a	n.a	n.a	6	–	–	3	–	1	10
KLO	1	9	–	–	–	–	–	–	–	10
KOP	14	–	–	–	–	–	2	1	–	17
MIL	4	3	–	–	–	–	1	3	–	11
MON	–	11	–	2	3	–	–	5	–	21
NIE	10	–	–	1	–	–	4	–	–	15
OBE	3	–	–	4	–	–	6	–	–	13
OST	n.a	n.a	n.a	8	–	–	17	1	–	26
WAL	n.a	n.a	n.a	9	–	–	2	1	–	12
WOE	3	1	2	–	–	–	2	3	–	11
WOL	2	4	–	1	–	–	–	–	–	7
ZEL	–	4	–	–	–	–	1	7	–	12
Total	54	43	2	36	3	0	41	23	1	203

n.a: not applicable

Median values of the TMRCA under the strict and uncorrelated lognormal molecular clock were quite similar: 1.302 × 10^6^ vs. 1.485 × 10^6^ years, whereby the highest posterior density (HPD) values ranged from just under 1 Myr to more than 2.3 Myr, in any case time periods that span over most if not all of the Pleistocene.

### Phenotypic analyses and correlation to genetic data

There was little allometry in morphological measurements globally, whereby the first two PCA factors accounted for over 78% of the global variance, yet neither factor was correlated with size. Closer inspection of individual variables showed a clear allometric pattern to eye size, determined primarily by the small-sized and large-eyed fish in ACH and to some degree KLO and NIE, and large-sized and small-eyed fish of the Baltic outgroup (OST) population. Eye measurements, however, did not load heavily on any PC factor. Much of the variance in the data set was determined by gill raker and lateral line scale counts (first two PCA factors) and body depth (third PCA factor). Nonetheless, we used the first six factors accounting for 90% of the total variance for our morphological distance matrix, as we aimed to include minor components of the global variance that may account for differentiation of single populations. The UPGMA dendrograms (Fig. S3, Supporting information) with the Baltic sample OST as outgroup (representing *C. maraena*), showed a clear split of the south Alpine KLO population, whereby the WOE population grouped with KLO when a higher threshold (0.90) was applied. The next split in both trees involved the hatchery reference (WAL). The remaining populations formed a cluster, in which there was some bootstrap support in both trees for MIL and WOL, whereby MIL is a rehabilitated population that has received brood from WOL.

The phenotypic distance matrix was correlated to both *F*_ST_-values (Spearman’s rho = 0.484, *P*= 0.021) as well as *R*_ST_ values (rho = 0.5361, *P*= 0.0103) when the 0.75 threshold for defining OU-native individuals was applied, whereby statistical significance was only substantiated for *F*_ST_ values when applying a 0.90 threshold (*R*_ST*,*_ Spearman’s rho = 0.384, *P*= 0.06; *F*_ST_, Spearman’s rho = 0.436, *P*= 0.026).

The coefficient of variance (CV) for all morphological traits of the OU-native was not correlated to mean population *q*-values when applying a 0.90 threshold. However, when using a threshold of 0.75 a negative trend was revealed, whereby KLO was the outlier representing an OU-native population with very high morphological variation. Removal of KLO resulted in significant negative correlations between the population CVs of all traits and mean *q*-values (Spearman’s rho: −0.527 to 0.673; *P*= 0.004–0.024).

## Discussion

The genetic structure of *Coregonus* in Austrian lakes reveals both ongoing and historical introgression with an introduced Baltic lineage. Introgression beyond the F1 generation is supported despite large credible intervals of assignment values, which do not allow the designation of individuals to backcross classes. The consequences of this introgression on the conservation potential of individual native stocks is highly variable and lake specific. Many lakes appear to harbour pure or nearly pure native genotypes despite the long history of regional stocking and only a few lakes appear to contain hybrid swarms. Thus, regionally, there are a number of lakes that should be given conservation priority, and shielded from introductions, a fact that was not considered prior to this study. For the lakes that do reveal non-native genotypes, a broad range of introgression intensity is assumed, most likely the result of the interaction between intrinsic isolating mechanisms and human-mediated ecological factors, long hypothesized to play a major role in promoting hybridization in freshwater fishes (Hubbs 1955). We pose that pre-zygotic mechanisms help maintain the integrity of native *Coregonus* stocks in the face of continued stocking, provided that the original stocks have not collapsed for unrelated (i.e. ecological) reasons. Additionally, the native Alpine lineage exhibits substantial geographic structure, which needs to be recognized by resource managers. This structure is concordant with the possibility that the native range of *Coregonus* extends to the south slopes of the Alps.

### Global recognition of OU-introduced and OU-native

structure analysis delineates two major lineages of *Coregonus* in Austrian lakes; the OU-introduced, commonly referred to as Maräne, and the OU-native. structure and newhybrids identified a similar number of OU-native individuals using a threshold of 0.90 whereby the number of OU-introduced detected by structure was much higher than that revealed by newhybrids. This differential result is not unusual (e.g. Cianchi *et al.* 2003; Burgarella *et al.* 2009) and is dependent on the proportion of hybrids and the number of diagnostic markers used (Vähä & Primmer 2006). Our results conform well with the prediction of Vähä & Primmer (2006) concerning the lower efficiency and higher accuracy of newhybrids compared to structure when the proportion of hybrids is low, which is the situation in a number of our lakes. The change in hybrid assignment efficiency in our data as a result of a lower threshold (0.75) is graphically depicted in Fig. S2 (Supporting information).

The two lineages (OU-native and OU-introduced) were not distinguishable with mtDNA data because the major clades were randomly distributed within the OU-native lineage among lakes. The TMRCA estimate supports that the two major clades (A and R) have a much older origin than the colonization of Central Europe, which was post-glacial (approximately 20 000 years ago) as all sampled lakes lie in previously glaciated regions. Thus we assume that the mtDNA lineages were already admixed prior to post-glacial expansion and have subsequently sorted out randomly among lakes, similar to the scenario reported for Swiss lakes (Hudson *et al.* 2010).

### Mechanisms controlling differential patterns of hybridization and introgression

Our data, in combination with recent reports (see below) on Austrian *Coregonus* strongly suggest several operative mechanisms preventing complete admixture in most lakes. Of the three classes of anthropogenic hybridization (Allendorf *et al.* 2001), hybridization without introgression is not apparent in our data, and thus we can assume that hybrids are not sterile. We base this inference on several aspects of our data independent of the wide credibility intervals of *q*-values, which do not allow assignment of hybrids to a backcross class. Most importantly, three lakes (MIL, WOE and ZEL) are currently not stocked with OU-introduced although they were extensively managed with these stocks in the past. Our data reflect the lack of pure OU-introduced individuals and thus there can be no F1 hybrids as the parental OU-introduced is absent. The *q*-value distributions in these three lakes thus reflect introgressed or hybrid swarm populations (Fig. 3). Further, our populations reveal little to no gametic disequilibrium, a state which should be prevalent in a population with F1 hybrids or rather recent admixture (Allendorf *et al.* 2001). This supports that admixture has been taking place for many generations. Lastly, unpublished experimental data show no sign of hybrid depression in the form of fertilization or hatching success in lab crosses. This supports the expectation that F1 hybrids should be able to further spread their genes through backcrossing. However, this does not exclude the possibility of other pre- or post-zygotic isolating mechanisms. For example, Pamminger-Lahnsteiner *et al.* (2009) reported temporal spawning segregation between the OU-native and OU-introduced lineages and Wanzenböck *et al.* (in press) experimentally demonstrated that these differences are under genetic control and thus may limit the number of opportunities to hybridize. Such a quantitative limitation alone should not prevent the development of a hybrid swarm given enough time and continued exposure (ongoing stocking) to the introduced lineage.

The distribution of admixture probabilities (*q*-values) for MON, FUS, NIE and OBE (Fig. 3) reflect ongoing hybridization and introgression but not complete admixture, despite ongoing introductions with the OU-introduced. Intuitively, higher stocking intensities should play a role in promoting a hybrid swarm. The Federal Fisheries Institute (FFI) on Mondsee has stocked between 28 and 542 fingerlings per hectare (mean 291) continuously from 1975 through 2009, while an additional not quantified number of sub-adult Maräne (ca. 20 cm) are also stocked each year (Jagsch, personal communication). Both the stocking of fingerlings as well as shipments of sub-adult Maräne to Mondsee has been both more continuous and more intense than any other lake under the oversight of the FFI (Jagsch, personal communication). The historical data are too limited to statistically evaluate the role of stocking intensity on our observed patterns of hybridization and introgression. However, stocking intensity alone cannot predict the level of introgression as one of the most intensely stocked lakes (e.g. MON) clearly does not display a hybrid swarm. If stocking intensity is poorly correlated to hybridization frequency then other factors influencing among lake differences, such as variable pre-zygotic isolating mechanisms or historical contingency must exist.

Several lakes have been reported to have lost their native stocks or experienced near extirpation through ecological degradation (Table 1). These lakes have been largely rehabilitated through development of wastewater control and treatment, and their fisheries rebuilt through stocking. ZEL is an example (Steyskal 2009), whereby several sources of brood fish were used during rehabilitation measures, and since 1970 only brood fish from the lake itself are used. The distribution of admixture proportions from ZEL, along with several lakes south of the Alps, namely MIL and WOE are more linear, indicative of a hybrid swarm. These results are consistent with a lack of isolating mechanisms due to either the rarity of one lineage (Rhymer & Simberloff 1996) or shifts in habitat heterogeneity or disturbance (Seehausen *et al.* 2008), such as the documented increase in eutrophication (see Table 1). The role of such ecological degradation in promoting introgression in lake coregonids has been most recently reported for Lake Thun in Switzerland (Bittner *et al.* 2010). In contrast to MIL, WOE and ZEL, lakes reported to have lower levels of historical eutrophication but nonetheless subject to stocking with OU-introduced (i.e. WOL, FUS and HAL) reveal limited to no signs of introgression, but indeed some individuals assigned to WAL. Stocking had ceased in HAL in the mid-1980s, only to be re-initiated in the last several years.

While we primarily base all inferences concerning hybridization on the microsatellite data, the mtDNA provides additional insights. The OU-introduced carries almost exclusively North European haplotypes (i.e. clade A), whereas hybrids carry at least some of the diversity exhibited by OU-native. Further, the mtDNA haplotype distribution in hybrid individuals supports that at least some hybrids stem from female native fish that have mated with introduced males.

The morphological correlation analysis provides an interesting assessment of the effect of a differing threshold (0.90 vs. 0.75) for defining hybrids. Clearly, individuals with some hybrid background, reflected by increased morphological CVs were assigned to the OU-native when using a threshold of 0.75. However, a threshold of 0.90 increased the efficiency of assigning hybrids, and thus there was no remaining correlation between morphological CVs of OU-native individuals and the level of introgression present in a lake.

### Genetic and phenotypic characterization of OU-native Coregonus populations in Austrian lakes

The sampled OU-native populations belong to three Danubian tributary systems, namely the Inn (ACH, NIE, OBE and ZEL), the Drau south of the Alps (MIL, KLO and WOE), and the Traun (all remaining samples). Within the large Inn drainage, ZEL, NIE and OBE are all in a major sub-drainage (the Salzach) quite distant from ACH, and are furthermore all heavily introgressed (Fig. 3). Thus, the distant genetic relationship of ACH to all others in the Inn drainage (Fig. 2) is easily explained. The within-lake structure (Fig. 3) for ACH appears similar to other putatively native populations, although it did receive some introductions from a foreign source in the seventies (Table 1). KLO, with no record of introductions, exhibits admixture probabilities indicating a pure native lineage yet lies outside the oft-cited natural distribution of *Coregonus* (Steinmann 1951). Honsig-Erlenburg & Mildner (1996) translated (from Latin) an historical text strongly suggesting that *Coregonus* is native to several Drau drainage lakes. The text stems from around 1800, but is based on citations reaching back into the mid 16th century whereby the name *Salmo Ošemize* was used, with *Ošemize* corresponding to the contemporary common Slavic name for *Coregonus*.

**Fig. 5:**
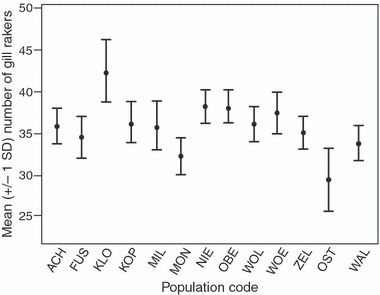
Mean gill-raker counts ± 1 standard deviation for 13 samples including the OU-introduced reference (WAL) and one wild caught Baltic sample (OST).

Furthermore, KLO individuals exhibit an atypical number of gill rakers, with a mean of 43 (SD 3.9), while the means of all other populations ranged from 32 to 38 (SD from 2.2 to 3.5; Fig. 5) excluding the possibility that these fish stem from contemporary stocking from any population examined in this study. If we can infer that *Coregonus* is indeed native to some lakes south of the Alps, then it is logical to assume that they were also found in the WOE population, one of the largest lakes in the Drau drainage. WOE contained a number of novel haplotypes genealogically related to the common haplotype in KLO, as well as several common private microsatellite alleles (data not shown) providing at least some evidence of their possible common ancestry.

The Traun drainage is structured into two sub-drainages, that of the Ager and the Traun itself. FUS and MON are in the Ager drainage and together with WOL group closely together based on both microsatellites (Fig. 2) and mtDNA data (Table 3). WOL is part of the main Traun drainage to which HAL and KOP belongs, but is located in a distinct tributary system. Indeed, HAL and KOP are comparatively distantly related to WOL based on both microsatellite and mtDNA haplotypes.

The north shore of WOL lies just 6 km south of MON but there is no record of their connection. WOL currently lies at 538 m above sea level (a.s.l.) and MON at 480 m a.s.l., connected by a very narrow pass that reaches 620 m a.s.l. Early post-glacial evidence suggests that both WOL and MON where about 50 m higher than today, and nearby Attersee (downstream from MON, at 469 m a.s.l.) was 132 m higher (Schadler 1959) implying a potential reverse flow to that found today. This speculative interpretation would explain the close genetic relation found between these two lake populations.

The KOP sample stems from the main tributary of HAL, where the lake’s native stocks are known to spawn (Haempel 1916; Lahnsteiner & Wanzenböck 2004). In this sample, neither hybrids nor introgressed individuals (similar to ACH and KLO) were detected suggesting either spatial segregation between the native and introduced lineages or the lack of successful reproduction for OU-introduced. A genetic screening of 35 larvae, caught in the lake (unpublished data) reveal no trace of the OU-introduced lineage.

## Conclusion

Our study of eight microsatellite loci, two concatenated mtDNA segments and various morphological traits in *Coregonus* sp. of Austrian lakes provided insights into the extent of hybridization and introgression between the native and an introduced Baltic lineage as well as the genetic structure of native populations. We were able to identify these two major lineages and their corresponding hybrids, albeit the pattern of population structure varied considerably among lakes. Indeed, we revealed different levels of hybridization and introgression and hypothesize that historical environmental degradation as well as other ecological factors like spawning segregation has played a role in the differential outcomes of introductions. Conservation planners need to be aware that generalizations concerning hybridization and introgression, even within a species, or in a specific region may be difficult to reach. Our identification of native populations and the extent of genetic effects by introductions of foreign stocks will be indispensable for effective conservation and management strategies in the region. We strongly urge managers to shield the remaining native gene pools from further invasion of non-native lineages, but also emphasize the notion that the maintenance of ecological integrity in ecosystems in general may be among the most important tools in shielding native fauna and flora from the potential negative effects of introduced species.

## Supporting information

**Table S1** Summary of microsatellite statistics for each sampled site. Shown are the sample sizes (*N*), number of alleles (*AN*), allelic richness (*AR*), allele size range (*R*), observed (*H_O_*) and expected (*H*_E_) heterozygosity, and *F*_IS_ values for all populations. ‘Overall’ lists the total number of alleles, the mean allelic richness, and heterozygosity and *F*_IS_ values calculated over all loci. An asterisk ‘*’ denotes a significant deviation from HWE based on 120 000 permutations and a table-wide alpha (0.05) correction

**Table S2** Accession numbers of all novel haplotypes (concatenated) used in this study. Asterisks mark partial sequences that already exist in Østbye *et al.* (2005)

**Table S3** Individual haplotypes observed in each lake, stemming from the concatenated sequences of the NADH-3 and cyt *b* mtDNA genes. Bold haplotypes are those observed in the present dataset as well as in Østbye *et al.* (2005); novel haplotypes are X1–X17 and all remaining haplotypes were found in Østbye *et al.* (2005) only

**Fig. S1** Ten biometric measurements and nine meristic characters were assessed. Morphological measurements were taken using an electronic calliper (+/− 0.1 mm) and included snout length (SNL 1–6), eye diameter (EYDIA 6–7), pectoral fin length (PVL 19–20), maxillary length (MXL 1–8), body depth (BDD 11–12), peduncle height (PDH 13–14), the inter orbital width (IOW – not shown), pectoral fin length (PCL 9–10), dorsal fin length (DOL 15–16), pelvic fin length (PVL 17–18), and anal fin length (ANL 19–20). Meristic characters, counted on the left side of specimens were the number of lateral line scales, scales above and below the lateral line, dorsal fin rays, pelvic fin rays, pectoral fin rays and the total number of gill rakers as well as the number on the upper and lower gill arch. For these latter counts the entire first gill arch was dissected and gill rakers counted under a dissecting microscope including all rudimentary rakers.

**Fig. S2** Bivariate plot of the first two dimensions from the factorial correspondence analysis (genetix 4.05) based on individual microsatellite genotypes. Individuals are colour coded: red for OU-introduced, green for OU-native and blue for hybrids according to their group assignment from structure: (A) threshold *q*-value 0.90 and (B) threshold *q*-value 0.75.

**Fig. S3** UPGMA dendrograms based on a Euclidean distance matrix of the mean population PCA scores from 10 morphological and 9 meristic measurements. Node support (1000 replicates) is shown when >50%. From Austrian lakes, only fish genetically assigned to OU-native with (A) threshold *q*-values >0.90 and (B) threshold *q*-values >0.75 were used. The Baltic (OST) sample serves as an outgroup. Cophenetic correlation = 0.87.
