# Supplementary material for: Hybridization and restricted gene flow between native and introduced stocks of Alpine whitefish (Coregonus sp.) across multiple environments
Source: Mol Ecol. 2011 Feb;20(3):456–72. doi: 10.1111/j.1365-294X.2010.04961.x (PMC3045663; doi:10.1111/j.1365-294X.2010.04961.x)
Supplement: Supplementary file 1 [file mec0020-0456-SD1.doc]

**Table S1** Summary of microsatellite statistics for each sampled site. Shown are the sample sizes (*N*), number of alleles (*AN*), allelic richness (*AR*), allele size range (*R*), observed (*H*_O_) and expected (*H*_E_) heterozygosity, and *F*_IS_ values for all populations. “Overall” lists the total number of alleles, the mean allelic richness, and heterozygosity and *F*_IS_ values calculated over all loci. An asterisk “*” denotes a significant deviation from HWE based on 120, 000 permutations and a table-wide alpha (0.05) correction.

| Sample site | *N* |  | ClaTet1 | ClaTet5 | ClaTet6 | ClaTet9 | ClaTet12 | ClaTet13 | ClaTet15 | ClaTet18 | Overall |
| --- | --- | --- | --- | --- | --- | --- | --- | --- | --- | --- | --- |
| FUS | 52 | *AN* | 11 | 15 | 15 | 8 | 28 | 8 | 8 | 6 | 99 |
|  |  | *AR* | 10.8 | 14.1 | 15.0 | 8.0 | 28.0 | 7.4 | 8.0 | 10.8 | 12.8 |
|  |  | *R* | 166-229 | 112-252 | 168-269 | 126-201 | 135-319 | 225-269 | 163-204 | 296-375 | - |
|  |  | *H*_O_ | 0.714 | 0.920 | 0.775 | 0.659 | 0.925 | 0.380 | 0.561 | 0.245 | 0.647 |
|  |  | *H*_E_ | 0.719 | 0.858 | 0.772 | 0.704 | 0.930 | 0.477 | 0.600 | 0.349 | 0.676 |
|  |  | *F_I_*_S_ | 0.017 | -0.062 | 0.009 | 0.077 | 0.018 | 0.213 | 0.078 | 0.308 | 0.054 |
| HAL | 40 | *AN* | 10 | 13 | 10 | 8 | 27 | 9 | 10 | 13 | 100 |
|  |  | *AR* | 8.2 | 10.1 | 9.2 | 7.9 | 23.1 | 7.5 | 10.0 | 11.9 | 11.0 |
|  |  | *R* | 174-229 | 120-252 | 168-269 | 134-201 | 135-306 | 217-265 | 127-280 | 276-375 | - |
|  |  | *H*_O_ | 0.750 | 0.650 | 0.667 | 0.910 | 1.000 | 0.534 | 0.783 | 0.853 | 0.769 |
|  |  | *H*_E_ | 0.750 | 0.828 | 0.817 | 0.800 | 0.945 | 0.581 | 0.758 | 0.843 | 0.790 |
|  |  | *F_I_*_S_ | 0.011 | 0.227 | 0.197 | -0.127 | -0.043* | 0.086 | -0.010 | 0.003 | 0.041 |
| KOP | 54 | *AN* | 10 | 9 | 6 | 7 | 28 | 6 | 11 | 13 | 90 |
|  |  | *AR* | 9.6 | 9.0 | 5.9 | 6.8 | 26.4 | 5.7 | 10.1 | 12.3 | 11.0 |
|  |  | *R* | 170-233 | 132-173 | 188-228 | 134-201 | 135-362 | 217-265 | 167-280 | 276-375 | - |
|  |  | *H*_O_ | 0.702 | 0.476 | 0.521 | 0.788 | 0.792 | 0.471 | 0.823 | 0.880 | 0.682 |
|  |  | *H*_E_ | 0.680 | 0.779 | 0.591 | 0.756 | 0.924 | 0.506 | 0.749 | 0.759 | 0.718 |
|  |  | *F*_IS_ | -0.014 | 0.420* | 0.159 | -0.066 | 0.151* | 0.056 | -0.088 | -0.149 | 0.061 |
| MON | 109 | *AN* | 9 | 14 | 15 | 10 | 28 | 11 | 7 | 10 | 104 |
|  |  | *AR* | 8.5 | 13.5 | 13.0 | 9.7 | 28.0 | 10.0 | 6.8 | 8.2 | 12.2 |
|  |  | *R* | 166-229 | 100-252 | 180-294 | 126-197 | 135-315 | 217-269 | 127-280 | 276-375 | - |
|  |  | *H*_O_ | 0.727 | 0.812 | 0.689 | 0.737 | 0.952 | 0.489 | 0.382 | 0.439 | 0.620 |
|  |  | *H*_E_ | 0.764 | 0.869 | 0.760 | 0.703 | 0.903 | 0.532 | 0.386 | 0.252 | 0.680 |
|  |  | *F*_IS_ | 0.053 | 0.070 | 0.099 | 0.051 | 0.059 | 0.087 | 0.018 | 0.429* | 0.094* |

| Lake | N(tot) |  | *ClaTet1* | *ClaTet5* | *ClaTet6* | *ClaTet9* | *ClaTet12* | *ClaTet13* | *ClaTet15* | *ClaTet18* | Overall |
| --- | --- | --- | --- | --- | --- | --- | --- | --- | --- | --- | --- |
| NIE | 32 | *AN* | 6 | 10 | 13 | 11 | 18 | 9 | 6 | 8 | 81 |
|  |  | *AR* | 5.3 | 7.9 | 10.3 | 10.0 | 13.8 | 6.7 | 6.0 | 7.0 | 8.4 |
|  |  | *R* | 174-229 | 120-252 | 172-278 | 134-238 | 127-290 | 296-375 | 167-280 | 296-375 | - |
|  |  | *H*_O_ | 0.700 | 0.968 | 0.654 | 0.857 | 0.793 | 0.633 | 0.333 | 0.567 | 0.688 |
|  |  | *H*_E_ | 0.701 | 0.820 | 0.723 | 0.854 | 0.908 | 0.687 | 0.656 | 0.629 | 0.748 |
|  |  | *F*_IS_ | 0.029 | -0.164 | 0.15 | 0.020 | 0.144 | 0.095 | 0.517* | 0.116 | 0.101 |
| OBE | 36 | *AN* | 8 | 15 | 14 | 8 | 26 | 11 | 9 | 8 | 99 |
|  |  | *AR* | 7.5 | 13.4 | 13.4 | 7.9 | 22.0 | 11.0 | 9.0 | 8.0 | 11.5 |
|  |  | *R* | 166-229 | 100-177 | 172-294 | 134-238 | 135-315 | 217-257 | 163-280 | 276-375 | - |
|  |  | *H*_O_ | 0.943 | 1.000 | 0.630 | 0.862 | 0.914 | 0.840 | 0.760 | 0.640 | 0.823 |
|  |  | *H*_E_ | 0.765 | 0.879 | 0.776 | 0.823 | 0.930 | 0.765 | 0.746 | 0.700 | 0.798 |
|  |  | *F_I_*_S_ | -0.218 | -0.123* | 0.206 | -0.029 | 0.032 | -0.078 | 0.002 | 0.106 | -0.014 |
| WOL | 29 | *AN* | 11 | 11 | 9 | 10 | 22 | 6 | 11 | 6 | 86 |
|  |  | *AR* | 9.5 | 10.3 | 8.6 | 8.9 | 22.0 | 4.5 | 8.5 | 4.7 | 9.6 |
|  |  | *R* | 166-245 | 112-193 | 188-299 | 126-201 | 131-306 | 225-257 | 167-288 | 296-367 | - |
|  |  | *H*_O_ | 0.828 | 0.815 | 0.826 | 0.643 | 0.947 | 0.172 | 0.621 | 0.172 | 0.628 |
|  |  | *H*_E_ | 0.803 | 0.870 | 0.754 | 0.696 | 0.928 | 0.194 | 0.616 | 0.223 | 0.636 |
|  |  | *F*_IS_ | -0.014 | 0.083 | -0.073 | 0.095 | 0.006 | 0.128 | 0.010 | 0.245 | 0.032 |
| ZEL | 39 | *AN* | 11 | 11 | 13 | 10 | 21 | 8 | 9 | 7 | 90 |
|  |  | *AR* | 9.9 | 10.4 | 12.4 | 9.2 | 19.9 | 7.5 | 9.0 | 6.4 | 10.6 |
|  |  | *R* | 166-245 | 112-193 | 168-299 | 134-388 | 135-319 | 225-257 | 127-288 | 296-367 | - |
|  |  | *H*_O_ | 0.811 | 0.737 | 0.823 | 0.818 | 0.968 | 0.703 | 0.963 | 0.517 | 0.783 |
|  |  | *H*_E_ | 0.768 | 0.852 | 0.857 | 0.811 | 0.932 | 0.732 | 0.808 | 0.444 | 0.785 |
|  |  | *F*_IS_ | -0.042 | 0.149 | 0.054 | 0.006 | -0.022 | 0.054 | -0.174 | 0.154 | 0.017 |
| KLO | 32 | *AN* | 7 | 8 | 12 | 7 | 8 | 2 | 5 | 5 | 54 |
|  |  | *AR* | 6.3 | 6.7 | 11.7 | 6.7 | 8.0 | 2.0 | 4.8 | 4.5 | 6.3 |
|  |  | *R* | 170-229 | 140-169 | 192-286 | 134-201 | 127-241 | 237-265 | 163-280 | 296-367 | - |
|  |  | *H*_O_ | 0.710 | 0.844 | 0.714 | 0.741 | 0.700 | 0.094 | 0.630 | 0.406 | 0.605 |
|  |  | *H*_E_ | 0.592 | 0.744 | 0.822 | 0.724 | 0.826 | 0.144 | 0.601 | 0.544 | 0.625 |
|  |  | *F*_IS_ | -0.183 | -0.118 | 0.155 | -0.005 | 0.178 | 0.363 | -0.029 | 0.269 | 0.052 |
| MIL | 58 | *AN* | 10 | 17 | 22 | 13 | 32 | 9 | 12 | 6 | 121 |
|  |  | *AR* | 9.9 | 16.0 | 20.5 | 12.3 | 32.0 | 8.2 | 11.6 | 5.6 | 14.5 |
|  |  | *R* | 166-245 | 112-252 | 184-290 | 126-388 | 106-311 | 221-269 | 127-293 | 296-375 | - |
|  |  | *H*_O_ | 0.891 | 0.920 | 0.717 | 0.863 | 0.810 | 0.339 | 0.800 | 0.200 | 0.692 |
|  |  | *H*_E_ | 0.857 | 0.846 | 0.898 | 0.800 | 0.941 | 0.372 | 0.783 | 0.203 | 0.713 |
|  |  | *F*_IS_ | -0.030 | -0.077 | 0.211* | -0.068 | 0.151* | 0.097 | -0.011 | 0.026 | 0.039 |

| Lake | N(tot) |  | *ClaTet1* | *ClaTet5* | *ClaTet6* | *ClaTet9* | *ClaTet12* | *ClaTet13* | *ClaTet15* | *ClaTet18* | Overall |
| --- | --- | --- | --- | --- | --- | --- | --- | --- | --- | --- | --- |
| WOE | 38 | *AN* | 18 | 14 | 21 | 9 | 28 | 9 | 12 | 10 | 121 |
|  |  | *AR* | 17.0 | 13.5 | 21.0 | 8.5 | 26.5 | 8.6 | 11.1 | 9.0 | 14.4 |
|  |  | *R* | 166-245 | 112-193 | 188-299 | 134-205 | 119-323 | 217-257 | 154-288 | 276-367 | - |
|  |  | *H*_O_ | 0.895 | 0.588 | 0.767 | 0.790 | 0.823 | 0.472 | 0.658 | 0.676 | 0.709 |
|  |  | *H*_E_ | 0.916 | 0.881 | 0.937 | 0.723 | 0.949 | 0.519 | 0.791 | 0.582 | 0.787 |
|  |  | *F*_IS_ | 0.036 | 0.346* | 0.198* | -0.078 | 0.147* | 0.104 | 0.181 | -0.146 | 0.114* |
| ACH | 40 | *AN* | 6 | 11 | 12 | 8 | 19 | 3 | 4 | 3 | 66 |
|  |  | *AR* | 5.8 | 10.5 | 12.0 | 7.8 | 19.0 | 2.9 | 3.7 | 2.9 | 8.1 |
|  |  | *R* | 158-182 | 120-181 | 188-409 | 134-201 | 153-298 | 229-241 | 127-293 | 280-300 | - |
|  |  | *H*_O_ | 0.622 | 0.730 | 0.867 | 0.842 | 0.900 | 0.412 | 0.278 | 0.118 | 0.596 |
|  |  | *H*_E_ | 0.612 | 0.651 | 0.803 | 0.794 | 0.911 | 0.333 | 0.305 | 0.112 | 0.565 |
|  |  | *F*_IS_ | -0.002 | -0.108 | -0.063 | -0.048 | 0.029 | -0.224* | 0.103 | -0.035* | -0.040 |
| KEL | 50 | *AN* | 9 | 13 | 8 | 8 | 19 | 13 | 7 | 8 | 85 |
|  |  | *AR* | 8.8 | 12.0 | 7.6 | 8.0 | 16.7 | 12.0 | 6.6 | 8.0 | 10.0 |
|  |  | *R* | 166-197 | 120-197 | 188-282 | 134-193 | 119-369 | 213-277 | 163-188 | 284-390 | - |
|  |  | *H*_O_ | 0.744 | 0.854 | 0.564 | 0.697 | 0.690 | 0.848 | 0.896 | 0.580 | 0.767 |
|  |  | *H*_E_ | 0.832 | 0.797 | 0.758 | 0.680 | 0.837 | 0.869 | 0.754 | 0.534 | 0.800 |
|  |  | *F*_IS_ | 0.119 | -0.059 | 0.268 | -0.010 | 0.187 | 0.035 | -0.178 | -0.071 | 0.044 |
| OST | 36 | *AN* | 8 | 9 | 16 | 10 | 21 | 10 | 10 | 9 | 93 |
|  |  | *AR* | 7.6 | 8.8 | 15.0 | 9.1 | 18.9 | 9.2 | 10.0 | 8.6 | 10.9 |
|  |  | *R* | 162-229 | 128-177 | 160-294 | 126-238 | 127-378 | 217--257 | 131-196 | 276-375 | - |
|  |  | *H*_O_ | 0.823 | 0.882 | 0.594 | 0.686 | 0.914 | 0.771 | 0.808 | 0.875 | 0.794 |
|  |  | *H*_E_ | 0.777 | 0.833 | 0.874 | 0.564 | 0.914 | 0.791 | 0.830 | 0.767 | 0.794 |
|  |  | *F*_IS_ | -0.078 | -0.088 | 0.283* | -0.225* | 0.018 | 0.052 | 0.050 | -0.130 | -0.001 |
| WAL | 102 | *AN* | 10 | 15 | 14 | 9 | 22 | 12 | 11 | 10 | 103 |
|  |  | *AR* | 9.7 | 14.3 | 13.5 | 9.0 | 21.4 | 11.5 | 11.0 | 9.1 | 12.4 |
|  |  | *R* | 166-229 | 112-252 | 176-294 | 134-238 | 119-294 | 182-269 | 154-280 | 296-375 | - |
|  |  | *H*_O_ | 0.693 | 0.730 | 0.786 | 0.787 | 0.720 | 0.907 | 0.803 | 0.650 | 0.759 |
|  |  | *H*_E_ | 0.740 | 0.648 | 0.847 | 0.799 | 0.870 | 0.873 | 0.830 | 0.696 | 0.788 |
|  |  | *F*_IS_ | 0.069 | -0.121 | 0.077 | 0.023 | 0.179* | -0.034 | 0.039 | 0.072 | 0.042 |

**Table S2** Accession numbers of all novel haplotypes (concatenated) used in this study. Asterisks mark partial sequences that already exist in Østbye *et al*. (2005).

| Novel haplotype | Accession number for the cytB segment | Accession number for the ND3 segment |
| --- | --- | --- |
| X1 | DQ173409* | HQ189767 |
| X2 | DQ173367* | HQ189768 |
| X3 | HQ189759 | DQ173352* |
| X4 | HQ189760 | DQ173414* |
| X5 | DQ173367* | HQ189769 |
| X6 | HQ189761 | DQ173354* |
| X7 | HQ189762 | DQ173414* |
| X8 | DQ173343* | HQ189770 |
| X9 | HQ189763 | DQ173354* |
| X10 | HQ189764 | DQ173414* |
| X11 | HQ189765 | DQ173414* |
| X12 | DQ173367* | HQ189770 |
| X13 | HQ189766 | DQ173354* |
| X14 | DQ173399* | HQ189771 |
| X15 | DQ173367* | DQ173354* |
| X16 | DQ173367* | DQ173414* |
| X17 | DQ173409* | DQ173414* |

**Table S3** Individual haplotypes observed in each lake, stemming from the concatenated sequences of the NADH-3 and Cyt *b* mtDNA genes. Bold haplotypes are those observed in the present dataset as well as in Østbye *et al*. (2005); novel haplotypes are X1-X17 and all remaining haplotypes were found in Østbye *et al*. (2005) only.

|  | Population | | | | | | | | | | | | | | | |  |
| --- | --- | --- | --- | --- | --- | --- | --- | --- | --- | --- | --- | --- | --- | --- | --- | --- | --- |
| Haplotype | ACH | FUS | HAL | KEL | KLO | KOP | MIL | MON | NIE | OBE | OST | TRA | WAL | WOE | WOL | ZEL | N |
| **A1** | 12 | 3 | 18 | 12 | 2 | 15 | 3 | 2 | 13 | 11 | 16 | 7 | 10 | 1 | 3 | 1 | 129 |
| A2 | - | - | - | - | - | - | - | - | - | - | - | - | - | - | - | - | 0 |
| A3 | - | - | - | - | - | - | - | - | - | - | - | - | - | - | - | - | 0 |
| A4 | - | - | - | - | - | - | - | - | - | - | - | - | - | - | - | - | 0 |
| **A5** | - | - | - | - | - | - | - | - | - | - | 10 | - | - | - | - | - | 10 |
| A6 | - | - | - | - | - | - | - | - | - | - | - | - | - | - | - | - | 0 |
| A7 | - | - | - | - | - | - | - | - | - | - | - | - | - | - | - | - | 0 |
| A8 | - | - | - | - | - | - | - | - | - | - | - | - | - | - | - | - | 0 |
| A9 | - | - | - | - | - | - | - | - | - | - | - | - | - | - | - | - | 0 |
| A10 | - | - | - | - | - | - | - | - | - | - | - | - | - | - | - | - | 0 |
| **A11** | - | - | - | - | - | - | 1 | - | - | - | - | - | - | 1 | - | - | 2 |
| A12 | - | - | - | - | - | - | - | - | - | - | - | - | - | - | - | - | 0 |
| A13 | - | - | - | - | - | - | - | - | - | - | - | - | - | - | - | - | 0 |
| A14 | - | - | - | - | - | - | - | - | - | - | - | - | - | - | - | - | 0 |
| A15 | - | - | - | - | - | - | - | - | - | - | - | - | - | - | - | - | 0 |
| A16 | - | - | - | - | - | - | - | - | - | - | - | - | - | - | - | - | 0 |
| B1 | - | - | - | - | - | - | - | - | - | - | - | - | - | - | - | - | 0 |
| B2 | - | - | - | - | - | - | - | - | - | - | - | - | - | - | - | - | 0 |
| C1 | - | - | - | - | - | - | - | - | - | - | - | - | - | - | - | - | 0 |
| C2 | - | - | - | - | - | - | - | - | - | - | - | - | - | - | - | - | 0 |
| D1 | - | - | - | - | - | - | - | - | - | - | - | - | - | - | - | - | 0 |
| E1 | - | - | - | - | - | - | - | - | - | - | - | - | - | - | - | - | 0 |
| F1 | - | - | - | - | - | - | - | - | - | - | - | - | - | - | - | - | 0 |
| F2 | - | - | - | - | - | - | - | - | - | - | - | - | - | - | - | - | 0 |

|  | Population | | | | | | | | | | | | | | | | |  |
| --- | --- | --- | --- | --- | --- | --- | --- | --- | --- | --- | --- | --- | --- | --- | --- | --- | --- | --- |
| Haplotype | ACH | FUS | HAL | KEL | KLO | KOP | MIL | MON | NIE | OBE | OST | TRA | WAL | WOE | WOL | ZEL | N | |
| F3 | - | - | - | - | - | - | - | - | - | - | - | - | - | - | - | - | 0 | |
| G1 | - | - | - | - | - | - | - | - | - | - | - | - | - | - | - | - | 0 | |
| H1 | - | - | - | - | - | - | - | - | - | - | - | - | - | - | - | - | 0 | |
| I1 | - | - | - | - | - | - | - | - | - | - | - | - | - | - | - | - | 0 | |
| I2 | - | - | - | - | - | - | - | - | - | - | - | - | - | - | - | - | 0 | |
| J1 | - | - | - | - | - | - | - | - | - | - | - | - | - | - | - | - | 0 | |
| K1 | - | - | - | - | - | - | - | - | - | - | - | - | - | - | - | - | 0 | |
| K2 | - | - | - | - | - | - | - | - | - | - | - | - | - | - | - | - | 0 | |
| L1 | - | - | - | - | - | - | - | - | - | - | - | - | - | - | - | - | 0 | |
| M1 | - | - | - | - | - | - | - | - | - | - | - | - | - | - | - | - | 0 | |
| M2 | - | - | - | - | - | - | - | - | - | - | - | - | - | - | - | - | 0 | |
| N1 | - | - | - | - | - | - | - | - | - | - | - | - | - | - | - | - | 0 | |
| N2 | - | - | - | - | - | - | - | - | - | - | - | - | - | - | - | - | 0 | |
| O1 | - | - | - | - | - | - | - | - | - | - | - | - | - | - | - | - | 0 | |
| O2 | - | - | - | - | - | - | - | - | - | - | - | - | - | - | - | - | 0 | |
| **P1** | - | - | - | - | 10 | - | - | 2 | - | - | 1 | - | - | - | - | - | 13 | |
| P2 | - | - | - | - | - | - | - | - | - | - | - | - | - | - | - | - | 0 | |
| P3 | - | - | - | - | - | - | - | - | - | - | - | - | - | - | - | - | 0 | |
| P4 | - | - | - | - | - | - | - | - | - | - | - | - | - | - | - | - | 0 | |
| P5 | - | - | - | - | - | - | - | - | - | - | - | - | - | - | - | - | 0 | |
| P6 | - | - | - | - | - | - | - | - | - | - | - | - | - | - | - | - | 0 | |
| Q1 | - | - | - | - | - | - | - | - | - | - | - | - | - | - | - | - | 0 | |
| Q2 | - | - | - | - | - | - | - | - | - | - | - | - | - | - | - | - | 0 | |
| Q3 | - | - | - | - | - | - | - | - | - | - | - | - | - | - | - | - | 0 | |
| **R1** | 1 | 10 | 5 | - | - | - | 9 | 16 | - | - | - | 3 | 1 | 3 | 4 | 12 | 64 | |
| R2 | - | - | - | - | - | - | - | - | - | - | - | - | - | - | - | - | 0 | |
| R3 | - | - | - | - | - | - | - | - | - | - | - | - | - | - | - | - | 0 | |
| R4 | - | - | - | - | - | - | - | - | - | - | - | - | - | - | - | - | 0 | |
| R5 | - | - | - | - | - | - | - | - | - | - | - | - | - | - | - | - | 0 | |
| **R6** | - | - | - | - | - | - | - | - | - | - | - | 1 | - | - | - | - | 1 | |
| S1 | - | - | - | - | - | - | - | - | - | - | - | - | - | - | - | - | 0 | |
| T1 | - | - | - | - | - | - | - | - | - | - | - | - | - | - | - | - | 0 | |
| T2 | - | - | - | - | - | - | - | - | - | - | - | - | - | - | - | - | 0 | |

|  | Population | | | | | | | | | | | | | | | |  |
| --- | --- | --- | --- | --- | --- | --- | --- | --- | --- | --- | --- | --- | --- | --- | --- | --- | --- |
| Haplotype | ACH | FUS | HAL | KEL | KLO | KOP | MIL | MON | NIE | OBE | OST | TRA | WAL | WOE | WOL | ZEL | N |
| U1 | - | - | - | - | - | - | - | - | - | - | - | - | - | - | - | - | 0 |
| U2 | - | - | - | - | - | - | - | - | - | - | - | - | - | - | - | - | 0 |
| U3 | - | - | - | - | - | - | - | - | - | - | - | - | - | - | - | - | 0 |
| U4 | - | - | - | - | - | - | - | - | - | - | - | - | - | - | - | - | 0 |
| X1 | 1 | - | 2 | - | - | 1 | - | - | - | - | - | - | - | - | - | - | 4 |
| X2 | - | - | 4 | - | - | 3 | - | - | - | - | - | 1 | - | - | - | - | 8 |
| X3 | - | - | 1 | - | - | - | - | - | - | - | - | 2 | - | - | - | - | 3 |
| X4 | - | - | - | 1 | - | - | - | - | - | - | - | - | - | - | - | - | 1 |
| X5 | - | - | - | 1 | - | - | - | - | - | - | - | - | - | - | - | - | 1 |
| X6 | - | - | - | - | - | - | 1 | - | - | - | - | - | - | - | - | - | 1 |
| X7 | - | - | - | - | - | - | - | 1 | - | - | - | - | - | - | - | - | 1 |
| X8 | - | - | - | - | - | - | - | - | 2 | - | - | - | - | - | - | - | 2 |
| X9 | - | - | - | - | - | - | - | - | - | 2 | - | - | - | - | - | - | 2 |
| X10 | - | - | - | - | - | - | - | - | - | - | - | 2 | - | - | - | - | 2 |
| X11 | - | - | - | - | - | - | - | - | - | - | - | 1 | - | - | - | - | 1 |
| X12 | - | - | - | - | - | - | - | - | - | - | - | - | 1 | - | - | - | 1 |
| X13 | - | - | - | - | - | - | - | - | - | - | - | - | - | 1 | - | - | 1 |
| X14 | - | - | - | - | - | - | - | - | - | - | - | - | - | 1 | - | - | 1 |
| X15 | - | - | - | - | - | - | - | - | - | - | - | - | - | 1 | - | - | 1 |
| X16 | - | - | - | - | - | - | - | - | - | - | - | - | - | 2 | - | - | 2 |
| X17 | - | - | - | - | - | - | - | - | - | - | - | - | - | 2 | - | - | 2 |
| Total | 14 | 13 | 30 | 14 | 12 | 19 | 14 | 21 | 15 | 13 | 27 | 17 | 12 | 12 | 7 | 13 | 253 |

**
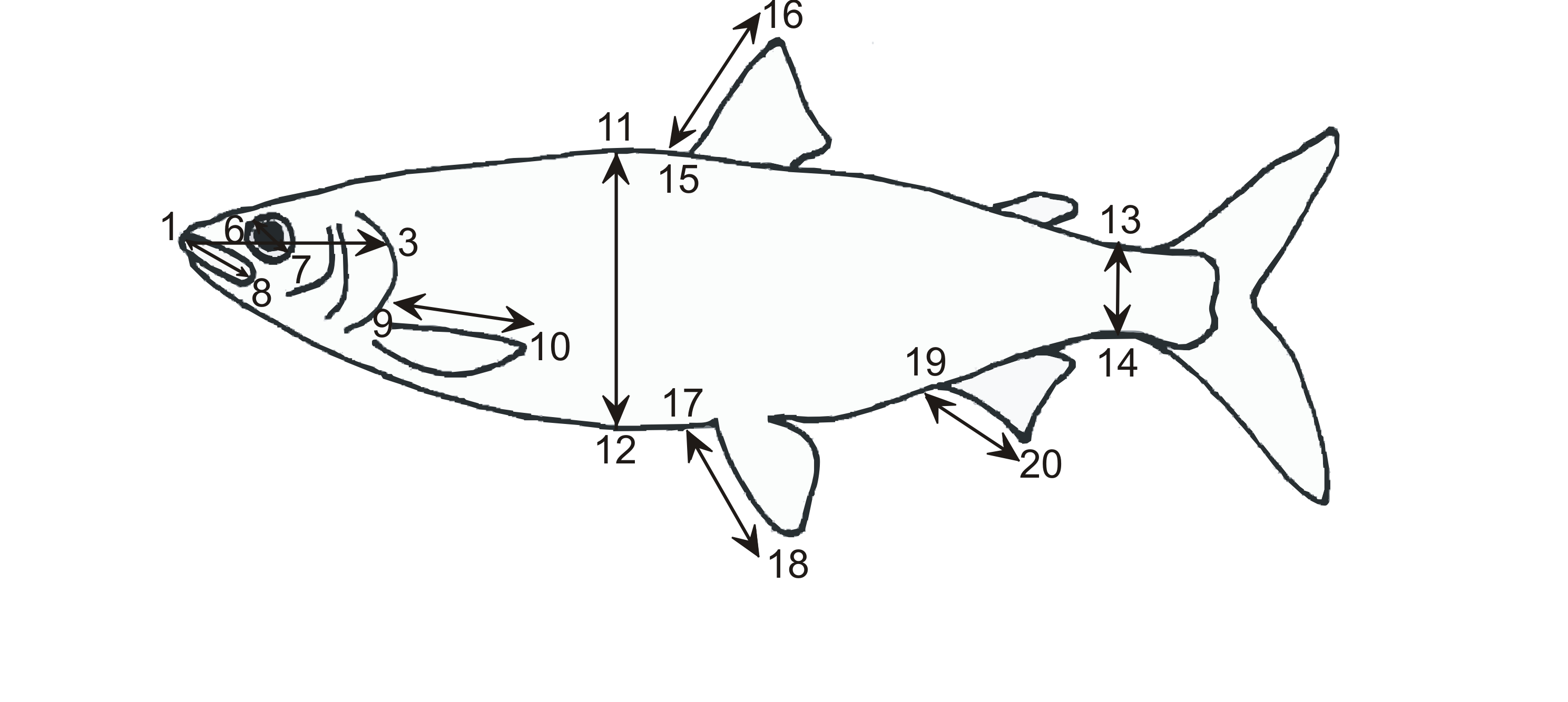
**

**Figure S1** Ten biometric measurements and 9 meristic characters were assessed. Morphological measurements were taken using an electronic calliper (+/-0,1mm) and included snout length (SNL 1-6), eye diameter (EYDIA 6-7), pectoral fin length (PVL 19-20), maxillary length (MXL 1-8), body depth (BDD 11-12), peduncle height (PDH 13-14), the inter orbital width (IOW – not shown), pectoral fin length (PCL 9-10), dorsal fin length (DOL 15-16), pelvic fin length (PVL 17-18), and anal fin length (ANL 19-20). Meristic characters, counted on the left side of specimens were the number of lateral line scales, scales above and below the lateral line, dorsal fin rays, pelvic fin rays, pectoral fin rays, and the total number of gill rakers as well as the number on the upper and lower gill arch. For these latter counts the entire first gill arch was dissected and gill rakers counted under a dissecting microscope including all rudimentary rakers.

**
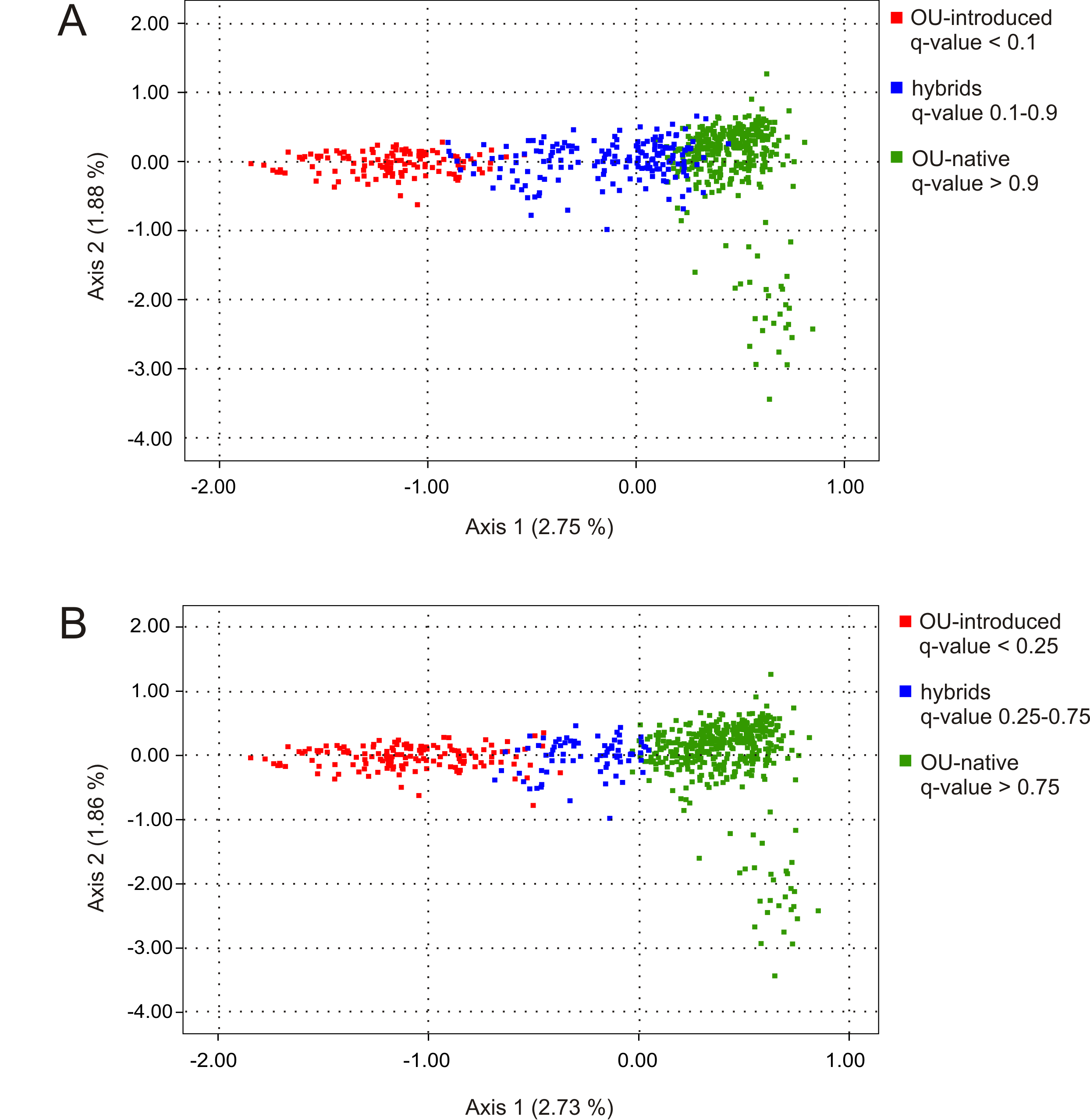
**

**Figure S2** Bi-variate plot of the first two dimensions from the factorial correspondence analysis (GENETIX 4.05) based on individual microsatellite genotypes. Individuals are color coded: red for OU-introduced, green for OU-native and blue for hybrids according to their group assignment from STRUCTURE: (A) threshold q-value 0.90 and (B) threshold q-value 0.75.

**
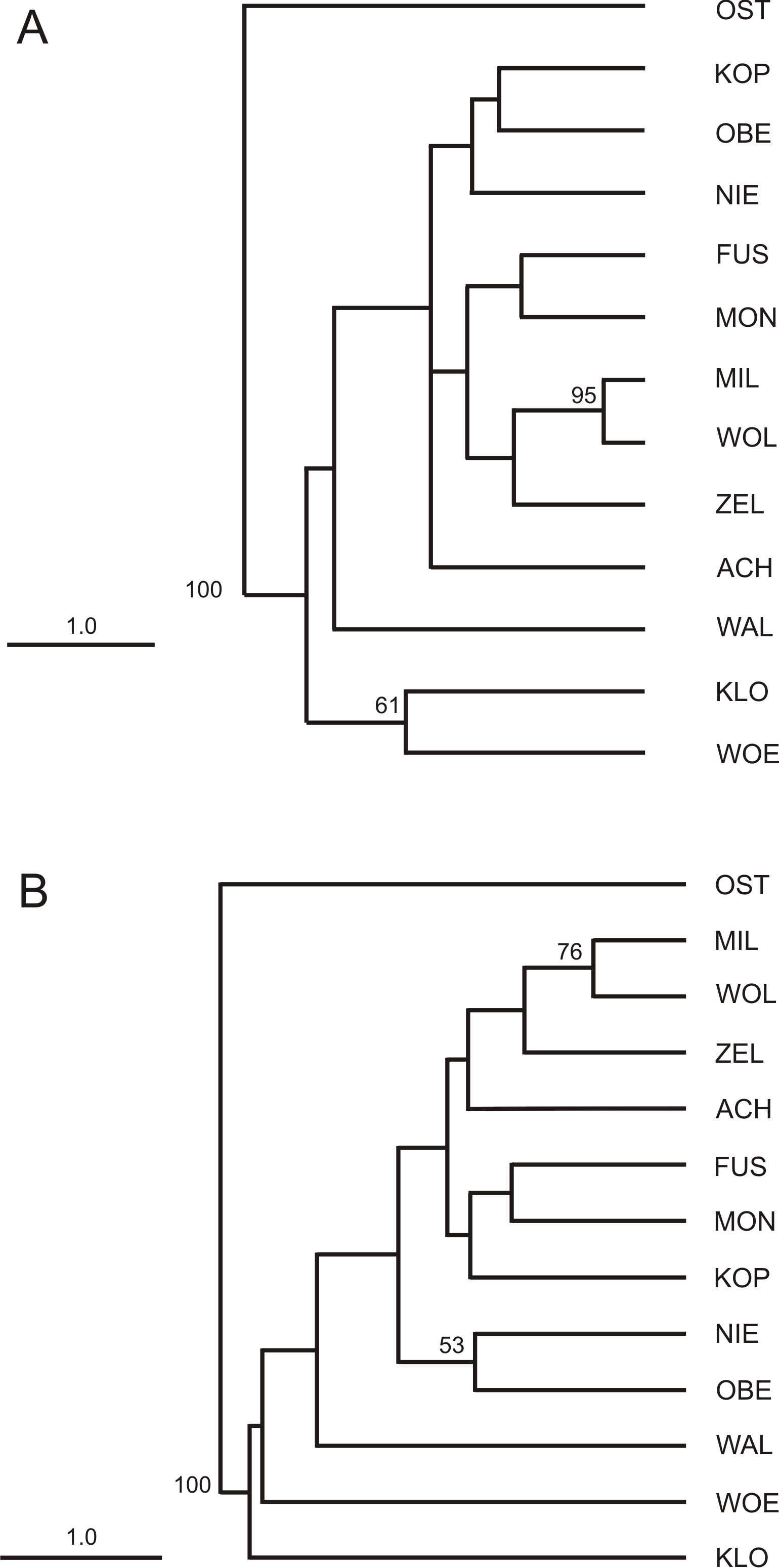
**

**Figure S3** UPGMA dendrograms based on a Euclidean distance matrix of the mean population PCA scores from 10 morphological and 9 meristic measurements. Node support (1000 replicates) is shown when greater than 50%. From Austrian lakes, only fish genetically assigned to OU-native with (A) threshold q-values >0.90 and (B) threshold q-values >0.75 were used. The Baltic (OST) sample serves as an outgroup. Cophenetic correlation = 0.87.
